# Postoperative Chylous Ascites in Gynecological Malignancies: Two Case Reports and a Literature Review

**DOI:** 10.1155/2024/1810634

**Published:** 2024-06-25

**Authors:** Xin Tan, GuoLin Luo, Guangdong Liao, Hong Liao

**Affiliations:** ^1^ Obstetrics and Gynecology Department West China Second University Hospital Sichuan University, Chengdu 610041, Sichuan, China; ^2^ Key Laboratory of Birth Defects and Related Diseases of Women and Children Sichuan University Ministry of Education, Chengdu 610041, Sichuan, China

## Abstract

**Aim:**

To explore the potential factors that influence the presentation and recovery of postoperative chylous ascites (CA) in gynecological malignancies.

**Methods:**

We reported two cases of postoperative CA following gynecological surgery and reviewed the clinical features of 140 patients from 16 relevant papers. Patients' clinicopathological characteristics, surgical approach, and management were summarized. The onset and resolution times of postoperative CA in different groups were analyzed separately.

**Results:**

The two patients in our report had recovery after conservative treatments. According to the literature review, the median time of onset of postoperative CA was 5 days (range, 0–75 days) after surgery. The median resolution time was 9 days (range, 2–90 days). Among patients, 87.14% of them had lymphadenectomy during gynecological surgeries, while 92.86% of the patients had resolution after conservative treatments.

**Conclusions:**

Lymphadenectomy during surgery may be relevant to the postoperative CA. Conservative management could be the initial choice for postoperative CA treatment.

## 1. Introduction

Chylous ascites (CA) is a rare condition caused by disruption of the lymphatic system, with the accumulation of milky triglyceride-rich chyle in the peritoneal cavity. Multiple etiologies have been reported, including traumatic, congenital, infectious, neoplastic, postoperative, cirrhotic, or cardiogenic. In 1992, the incidence of CA at a large university-based hospital over a two-decade period was reported to be approximately 1 in 20,000 admissions [1]. The rate has likely increased in recent years due to improved survival rates among patients with cancer, extensive surgical interventions, as well as advances in laparoscopic surgery and transplantation [2].

The symptoms of CA are nonspecific. The most typical feature is abdominal distention (81%), followed by indigestion, nausea, and vomiting [3]. The severity depends on the amount of ascitic fluid and its accumulation rate, as well as the patient's health condition. Serious cases of peritonitis and ileus have also been reported. In some cases, deterioration with environmental disturbances and immunological dysfunctions have also been reported [3, 4].

Treatment strategies for postoperative CA are broadly divided into two categories, namely, conservative management (dietary restriction and medical therapy) and surgery [4]. Conservative management, which aims to reduce the production of chyle and promote closure of the fistula, is successful in most cases [5]. Surgical intervention is usually performed following unsuccessful conservative management [6].

Postoperative CA is infrequent after gynecological surgery. Most available studies are case reports. Experience in the prevention, diagnosis, and treatment of postoperative CA is lacking. Here, we described two cases of postoperative CA following gynecological surgeries and reviewed the relevant articles on patients with gynecological malignancies and postoperative CA. This study aimed to describe the clinical features of CA after gynecological surgery and to determine the potential factors associated with its prognosis.

### 1.1. Case 1

**Figure 1:**
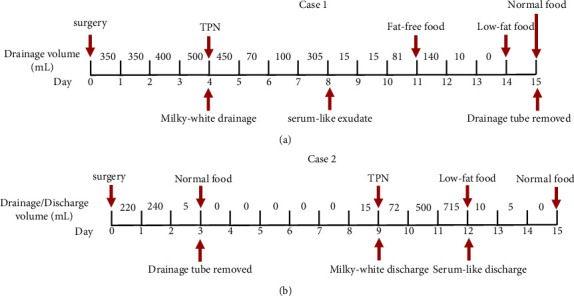
The postoperative conservative management and the daily drainage/discharge volume for patients in case 1 (a) and case 2 (b). TPN: total parenteral nutrition.

The first case was a 14-year-old girl with a large pelvic mass. A large complex mass in the abdominal cavity was observed on ultrasonography, and a plain MRI scan confirmed a 31 cm × 25 cm × 16 cm cystic-solid mass with calcification and composed of fat, enwrapped with the omentum. Several serum tumor markers were increased. In particular, CA125 was 202.84 U/mL and CA19-9 was 98.61 U/mL. The alpha-fetoprotein (AFP) level was 262.04 ng/mL. The patient was subsequently admitted to our hospital for surgical treatment, and open surgery was performed immediately. A 30 cm × 25 cm × 16 cm mass, weighing 7.5 kg, was removed. The mass originated from the right ovary and densely adhered to the peritoneum and intestine. The common iliac and para-aortic lymph nodes were unusually enlarged and fused into one large mass of approximately 15 cm × 8 cm × 7 cm, which was adhered to the aorta and inferior vena cava. A Müllerian anomaly was also observed with a small rudimentary horn of the uterus in the right pelvic cavity. During surgery, the ovarian mass along with the right fallopian tube, enlarged lymph nodes, and the right rudimentary horn of the uterus were carefully removed. Grossly, the solid mass consisted of separate cysts with necrotic and hemorrhagic contents. Small amounts of hair and yellow fat were also observed inside the mass. The microscopic appearance and immunohistochemical results confirmed that the mass consisted of two major parts: a yolk sac tumor and a mature teratoma, both originated from ovarian germ cells. After surgery, the abdominal drainage was normal. A clean yellow liquid (approximately 350–400 mL per day in the first three days) was drained. In addition to an intravenous infusion, the patient was also provided with soft digestible food to eat. On postoperative day 4, drainage increased to 500 mL per day and the color changed to milky white. The drained fluid was analyzed: red blood cell count, 1.7 × 10^9^/L; white blood cell count, 1.5 × 10^7^/L; cholesterol, 1.65 mmol/L; total protein, 0.178 g/L; albumin, 0.06 g/L; triglycerides, 4.44 mmol/L; amylase, 50 U/L; and cultures were negative. The patient was only slightly hypodynamic. Food was restricted once CA was diagnosed, but water was taken at will. Parenteral nutrition (PTN) was provided through peripheral vein infusion to guarantee daily nutrients and calories (25 kcal/kg plus 10% extra calories per day). One compound nutrient formulation containing 885 mL of 11% glucose, 300 mL of essential amino acids (34 g), and 255 mL of 20% intralipid solution supplied 1000 kcal. Moreover, 1000 mL of 10% glucose solution supplied 400 kcal and 500 mL of 8.5% compound amino acid solution supplied 42.5 g amino acids. The daily drainage volume and appearance are shown in Figure 1(a). Fat-free semi-liquid food was given from day 11 and then changed to low-fat soft food on day 14. The patient resumed her normal diet on day 15 postsurgery. The drainage tube was removed on day 15. Subsequently, the patient began chemotherapy. To date, the patient has received six cycles of bleomycin, etoposide, and platinum (BEP) chemotherapy and is still being followed.

### 1.2. Case 2

The second patient was a 55-year-old woman who underwent supracervical hysterectomy for a uterine tumor 4 years ago. Histopathological examination revealed that the tumor was low-grade endometrial stromal sarcoma. The patient refused further treatment until relapse occurred. Irregular vaginal bleeding persisted for 10 days. A mass, measuring 5 cm × 4 cm × 4 cm, was fixed to the residual cervix and palpated through a bimanual examination. Open surgery was performed to remove the mass, along with the residual cervix and bilateral ovaries. The resection of the parametrium was the same as that for radical hysterectomy. Tumor emboli in the small veins were found in the cardinal ligaments, sacral ligaments, and parametrium tissues. Pelvic and para-aortic lymphadenectomy was not performed because it would not have benefited the patient owing to the extensive metastatic tumor embolus. The final histopathological results confirmed the relapse of low-grade endometrial stromal sarcoma. Abdominal drainage was normal. A clean yellow liquid (approximately 220 mL–240 mL per day in the first three days) was drained. The drainage tube was removed on day 3 postsurgery. On day 9, a milky-white discharge from the patient's vaginal cuff when she sat up was detected. The uric acid, urea, and creatinine levels were 204 *μ*mol/L, 3.50 mmol/L, and 52 *μ*mol/L in the blood; 165 *μ*mol/L, 3.40 mmol/L, and 50 *μ*mol/L in the discharge; and 1190 *μ*mol/L, 53.60 mmol/L, and 10854 *μ*mol/L in the urine. Based on these results, the diagnosis of urinary fistula was excluded. The biochemistry of the drained fluid was also tested: red blood cell count, 0.9 × 10^9^/L; white blood cell count, 1.1 × 10^7^/L; cholesterol, 1.45 mmol/L; total protein, 0.16 g/L; albumin, 0.04 g/L; triglycerides, 4.21 mmol/L; amylase, 47 U/L; and cultures were negative. Treatment for this patient was similar to the first case. Food was restricted once CA was diagnosed. Nutrition was guaranteed through parenteral feeds via a peripheral vein. The patient was also provided with low-fat soft food to eat. The discharge decreased from day 12 and totally resolved on day 15. The patient's daily discharge and the conservative management plan are summarized in Figure 1(b). After systematic evaluation by a gynecological oncologist, the patient was administered letrozole for therapy.

## 2. Literature Review

We conducted a literature review to evaluate the risk of CA after gynecological surgery. We searched the following databases: PubMed, Embase, and the Cochrane Library. All relevant peer-reviewed articles with the keywords, “chylous ascites,” “gynecological surgery,” “chylous ascites,” and “gynecological malignancies” were considered. Time or language limitations were not included. The type of paper was also not limited.

### 2.1. Inclusion and Exclusion Criteria

All included studies met the inclusion criteria as follows: (1) full texts were available to identify the relevant information. (2) Patients who underwent gynecological surgeries for benign or malignant diseases. (3) The symptoms of CA were obvious. (4) Diagnosis of lymphatic ascites by clinical examination, abnormal abdominal drainage or vaginal discharge, abdominal paracentesis, CT, or ultrasonography.

The exclusion criteria were as follows: (1) patients who had received chemotherapy or radiotherapy between surgery and CA. (2) Patients who had ureteral or bladder injury during surgery. (3) Patients who had other identified causes of ascites including congenital disease, cardiac disease, liver disease, or malabsorption syndrome.

### 2.2. Data Retrieval

The publication year, characteristics of individual patients in each study including age, diseases, surgeries, lymph node metastasis, adjuvant therapy, onset, and resolution time of CA, as well as the treatment approach, were precisely extracted and recorded in a separate contingency table. All data were checked by two investigators before entering the data for analysis.

## 3. Results

A total of 16 papers met the inclusion and exclusion criteria and were included after a careful review of the full contents. In total, 140 patients were available for analysis [7–22]. Information on the patients' clinicopathological characteristics, surgical approach, and CA management are summarized in Tables 1 and 2.

The median age of the patients was 52 years (range, 18–83 years). All patients were diagnosed with malignant gynecological diseases, except for one patient with primary peritoneal cancer. Among them, 32 (22.86%) had cervical cancer, 46 (32.86%) had ovarian cancer, 55 (33.29%) had endometrial cancer, 5 (3.57%) had synchronous ovarian and endometrial cancer, and 1 (0.71%) had fallopian tube cancer. More than half of the patients (87 patients, 62.14%) underwent open surgery and 38 (27.14%) underwent laparoscopy, while the rest were not clear. For lymphadenectomy, most patients (122 patients, 87.14%) had bilateral pelvic and para-aortic lymph node dissection during surgery, 14 patients (10%) had pelvic lymph node dissection, and 3 patients (2.14%) had para-aortic lymph node dissection. In terms of lymph node (LN) status, 73 patients (52.14%) had no metastasis, 11 (7.86%) had only positive pelvic LN, 11 (7.86%) had only positive para-aortic LN, and 9 had both positive para-aortic and pelvic LN.

The onset of postoperative CA started 0–75 days after surgery, with a median of 5 days. The median resolution time was 9 days (range, 2–90 days). For conservative management, nearly a third of the patients (39 patients, 27.86%) were given a high-protein, low-fat, medium-chain triglyceride (MCT)-based diet. More than half of the patients (73 patients, 52.14%) received total parenteral nutrition (TPN), and 13 patients (9.29%) received both management strategies in sequence. Among the patients who received TPN, 21 received somatostatin as supplemental treatment. Finally, 10 patients underwent surgery to correct lymph leakage due to unsuccessful conservative management.

The patients were divided into different groups according to the primary site of disease, surgical approach, lymphadenectomy, lymph node status, conservative management, surgical treatment, and drug therapy. We analyzed the onset and resolution times of CA in these different groups. Only patients with specific grouping information and onset or resolution time were included in this analysis. The median onset and resolution times in each group are listed in Table 3.

## 4. Discussion

Chylous ascites is an infrequent postoperative complication. Among patients with gynecological malignancies, the prevalence ranges from 0.17% to 9% [14–16, 19–21]. Notably, numerous cases may be subclinical or may resolve spontaneously; thus, estimating the true prevalence is difficult. Moreover, most published studies are single-center studies. Therefore, the sample size, selection bias, and surgical procedure differences may all contribute to a different incidence rate. In Zhao's study, the prevalence rate was 0.9% in 997 patients who underwent laparoscopic surgery [19]. In contrast, the prevalence was 9% in Solmaz et al.'s study, which included 399 patients who underwent either open or laparoscopic surgery [20]. Moreover, the placement of a drainage tube was associated with a higher prevalence. In some studies, a peritoneal drainage tube was not routinely placed during surgery, which may have led to not identifying mild CA, resulting in a relatively lower incidence [14–16, 21]. Diagnosis is made based on the milky ascitic fluid, which contains triglycerides >200 mg/dL [5]. The ascitic fluid is often detected using an abdominal drainage tube or from paracentesis.

Intraoperative cisterna chili injury and retroperitoneal lymphatic gland dissection are believed to be the most common causes of CA [5]. Normally, a small amount of lymphatic leakage caused by lymphadenectomy resolves spontaneously without any symptoms. Injury to the cisterna chili or major lymphatic channels can result in the accumulation of lymphatic fluid in the abdominal cavity and lead to symptoms, such as distention, nutrient imbalance, and infection [5]. Cisterna chili receives fatty chyle from the intestine, and thus, its injury results in the accumulation of white or straw-colored lymphatic fluid in the abdominal cavity. This is a distinct clinical feature. Radiotherapy following surgery may also be associated with CA. Baiocchi et al. reviewed four English studies, including 31 cases, and found that 28 patients with CA underwent radiation therapy together with retroperitoneal lymph node dissection before the onset of CA [12]. This is probably due to radiation fibrosis of the lymphatic vessels in the small bowel, leading to occlusion and extravasation of the chylous fluid [23].

Lymphatic spread is one of the most common patterns of tumor dissemination in gynecological malignancies. According to the National Comprehensive Cancer Network (NCCN), bilateral pelvic lymph node dissection (PLND) and para-aortic lymph node dissection (PALND) are part of the surgical treatment of advanced gynecological malignancies [24–26]. The number of lymph nodes removed has been reported to be related to the incidence of CA. In the THIEL study, patients with CA had a higher mean number of removed lymph nodes than those who had not developed CA (51.9 vs. 40.0, *p*=0.002) [21]. In Tulunay's study, the median number of removed para-aortic lymph nodes (PALN) was 26 (range, 8–54) in patients with CA and 17 (range, 1–76) in patients without CA [16]. They suggested that the number of harvested PALN, other than the pelvic lymph nodes, was relevant to the development of CA, which was also consistent with Solmaz's findings. Solmaz et al. recommended an optimal cutoff value of >14 harvested PALNs in the prediction of CA, calculated using the receiver operating characteristic (ROC) curve analysis [20]. The dissection of para-aortic lymph nodes usually includes the region from the bifurcation of the aorta to the superior mesenteric artery or the renal veins and is more likely to damage the central lymphatics than the dissection of pelvic lymph nodes. The first patient in our study had a large para-aortic lymph node that was dissected using an ultrasonic device. Thermal injury to the surrounding tissues or even central lymphatics may be responsible for postoperative CA. In the second patient, the intra-abdominal adhesions resulting from the previous surgery were extremely severe, and the normal anatomical structure was severely damaged. Lymphatic vessels may have been injured, even though no lymph nodes were removed.

The surgical approach (laparoscopy and open surgery) may also be associated with postoperative CA. Divergent lymphatic vessel sealing approaches, harvesting techniques, differences in intra-abdominal pressure, and accessible lymph node regions may have been responsible for this discrepancy. Zhao et al. analyzed 997 patients who underwent laparoscopic lymphadenectomy and reported that only 9 patients (0.9%) developed CA [19]. This was a relatively low prevalence, but there was no control data for open surgery in their study to draw comparisons. In the THIEL study, 20 of 28 patients with CA (71.4%) had laparoscopic treatment. This was significantly more than those who underwent open surgery (8/28; 28.6%) (*p* < 0.0001) [21]. However, no other studies have compared the prevalence of CA after two different approaches, thus it remains inconclusive if one approach is superior to the other in preventing postoperative CA. In our analysis, laparoscopic patients had a shorter onset time than open surgery patients (3.5 days vs. 5 days), but the two groups had a similar resolution time (9 days vs. 8 days), suggesting that laparoscopic patients may receive an earlier diagnosis and treatment. This in turn, may have resulted in a shorter hospital stay and lower medical costs.

As ascitic fluids contain abundant metabolites of long-chain triglycerides, food restriction aimed to largely reduce the production of chyle [2, 27]. Thus, a high-protein and low-fat diet with MCT is recommended in practice [28]. If they do not respond well to MCT, patients are asked to fast and rely on total parenteral nutrition (TPN) [29]. In a large retrospective study, the effectiveness of TPN in patients with postoperative CA was 100% [30]. The MCT diet provided essential nutrients and was more accepted by the patients. However, summarized from 36 articles, Weniger et al. suggested TPN as the priority selection for patients who had chylous leakage of more than 200 mL/day. The MCT diet is a more suitable option for patients with chylous leakage of less than 200 mL/day or when TPN is contraindicated [30]. In one retrospective study, an enteral nutrition (EN) + MCT plan was reported as superior to TPN alone or MCT alone in terms of the curative effectiveness and cost of treating CA [31].

As for medical therapy, somatostatin, a peptide hormone that suppresses the secretion of numerous gastrointestinal hormones and gastric emptying when working together with TPN or MCT, contributes to relieve the symptoms of CA [31, 32]. Octreotide, a somatostatin analog, has also been demonstrated to be effective in refractory postoperative CA by relieving portal hypertension and reducing the triglyceride levels in ascites [18, 33]. In a small retrospective clinical analysis, octreotide plus TPN worked better than TPN alone in eliminating CA [34]. Thus, octreotide should be considered if TPN is administered to patients. According to a retrospective study, the application of somatostatin or its analog octreotide improved symptoms and shorten hospital stays [31].

Surgical intervention is usually performed if conservative management fails. The fistula could be ligated once located via lymphangiography or lymphoscintigraphy before surgery or identified during surgery [35, 36]. Less radical approaches, such as abdominal paracentesis, transjugular intrahepatic portosystemic shunt, and peritoneovenous shunts, are also alternatives [5]. Paracentesis promptly relieves distention and can help make a diagnosis, but repeated performance may lead to nutrient loss, hypoproteinemia, and increased risk of bacterial peritonitis [37]. Successes of lymphatic embolization following lymphangiography have also been widely reported [38, 39]. Lymphatic embolization could be achieved through different percutaneous approaches with different liquid embolic agents or balloons to stop the leakage. Normally, the resolution time in patients who underwent surgical treatment was longer than that in patients who underwent conservative management because surgery was not the first-line choice for patients. This was observed in our analysis, where the resolution time was 41 days in the surgical group and 9 days in the conservative group.

Generally, the prognosis of postoperative CA is better than that of nonoperative CA. In our analysis, the median time for resolution was 9 days, with a wide range of 2 to 90 days. Specifically, in Solmaz et al.'s study, the median time (5 days, 2–12 days) was the shortest [20]. However, the longest resolution time was reported by Boran, who reported a patient who had CA lasting for 90 days after para-aortic lymph node dissection [8]. This patient sequentially received TPN + somatostatin, repeated paracentesis, MCT + somatostatin, and underwent surgery to thoroughly correct the CA. Encouragingly, conservative management works well in patients with CA following gynecological surgeries, with a success rate of 100% in certain studies [15, 19, 20]. In our analysis, 92.86% of patients had a good prognosis after conservative management.

## 5. Conclusions

The current study consists of two parts. First, we reported two cases of postoperative CA caused by gynecological surgeries and described our experience with successful conservative treatment. Then, we reviewed 16 studies that included 140 patients with postoperative CA. Lymphadenectomy during surgery may be relevant to the occurrence of postoperative CA. Once diagnosed, conservative management could be the initial choice for postoperative CA treatment, and most patients could get resolution from it.

## Figures and Tables

**Table 1:** Clinicopathological characteristics, surgical information, and management of patients with chylous ascites.

Author	Year	Age	Primary site	Pathology	Stage	Major surgery	Lymphadenectomy	Surgical approach	Lymph node status		Chemotherapy or radiation	Onset (day)	Resolution (day)	Management		
									Positive pelvic lymph node	Positive para‐aortic lymph node				Diet	Drug	Surgery
Krishnan et al.	2001	NS	Cervix	NS	IB	RH + BSO	P	O	NS	NS	N	6	8	NS	NS	—
		NS	Cervix	NS	IB	RH + BSO	P	O	NS	NS	N	3	22	NS	NS	—
		NS	Endometrium	NS	IC	TAH + BSO	P	O	NS	NS	N	13	16	NS	NS	—
		NS	Cervix	NS	IB	RH + BSO	P	O	NS	NS	N	4	11	NS	NS	—
		NS	Cervix	NS	IB	RH + BSO	P	O	NS	NS	N	7	8	NS	NS	—
		NS	Cervix	NS	IB	RT + BSO	P	O	NS	NS	N	9	53	NS	NS	—
		NS	Ovary (recurrence)	NS	IIIC	—	PA	O	NS	NS	Y	3	NS	NS	NS	Y

Boran et al.	2004	35	Ovary	Mucinous borderline tumor	NS	TAH + BSO + O + A	BPPALND	O	—	—	N	11	90	MCT + TPN	Somatostatin	Y

Dewdney et al.	2006	19	Ovary	Immature teratoma	NS	Exploratory laparotomy; removal of ovarian mass	No	L	NS	NS	Chemotherapy	22	77	MCT	NS	—

Takeuchi et al.	2006	56	Cervix	Adenosquamous cell carcinoma	IIIB	RH + BSO	BPPALND	O	NS	NS	Chemotherapy	4	24	MCT + TPN	NS	—

Williams et al.	2007	49	Cervix	Squamous cell carcinoma	IBI	RH	P	L	—	—	N	22	42	MCT	Octreotide	—

Baiocchi et al.	2010	50	Ovary	Serous-papillary ovarian cancer	IIIC	TAH + BSO + O + A, resection of ileal and colonic implants	BPPALND	O	—	—	N	9	22	MCT + TPN	Octreotide	—
		63	Endometrium	Endometrioid adenocarcinoma	IBG3	TAH + BSO + O	BPPALND	O	—	—	N	4	10	MCT + TPN	Octreotide	—

Var et al.	2011	35	Cervix	Squamous cell cervical cancer	IB1	RH	BPPALND	L	NS	NS	Radiotherapy	20	42	MCT + TPN	Octreotide	—
		54	Endometrium	NS	IB	TAH + BSO	BPPALND	O	NS	NS	N	NS	14	MCT + TPN	Octreotide	—
		47	Endometrium	NS	IIA	RH	BPPALND	NS	NS	NS	N	5	10	MCT + TPN		—
		62	Ovary	Serous adenocarcinoma	NS	TAH + BSO	BPPALND	O	NS	NS	N	7	7	MCT + TPN	Octreotide	—

Han et al.	2012	51	Endometrium	Adenocarcinoma	IIIA	MRH + BSO	BPPALND	NS	—	—	N	17	18	NS	NS	—
		64	Cervix	Squamous cell carcinoma	IIA	RH + BSO	BPPALND	NS	—	—	N	35	3	NS	NS	—
		55	Ovary (recurrence)	Adenocarcinoma	IIIC	Part small-bowel resection and part omentum resection	PA	NS	—	Y	Chemotherapy	75	28	NS	NS	—
		41	Cervix	Squamous cell carcinoma	IA2	RH	P	NS	—	—	N	69	17	NS	NS	—
		56	Cervix	Adenocarcinoma	IA	TAH + BSO + O + A	BPPALND	O	—	—	N	5	3	NS	NS	—
		56	Cervix	Squamous cell carcinoma	IB1	RH + BSO	P	NS	—	—	N	7	21	NS	NS	—
		40	Ovary	Adenocarcinoma	IIIC	TAH + BSO + O, abdominal and pelvic cytoreductive surgery	PA	O	—	Y	N	5	3	NS	NS	—

Tulunay et al.	2012	66	Endometrium	Endometrioid	Ia	TAH + BSO + O	BPPALND	O	—	—	N	25	13	MCT	—	—
		63	Endometrium	Endometrioid	Ia	TAH + BSO + omental biopsy	BPPALND	O	—	—	N	5	7	TPN	—	—
		36	Ovary	Borderline serous tumor	IIIc	TAH + BSO + O	BPPALND	O	Y	—	N	22	16	TPN	—	Y
		53	Cervix	Adenocarcinoma	Ib1	—	BPPALND	NS	Y	Y	N	5	10	TPN	—	—
		58	Endometrium	Serous adenocarcinoma	IVb	TAH + BSO + O	BPPALND	O	—	—	N	7	29	TPN	—	—
		50	Ovary	Clear-cell carcinoma	Ic	TAH + BSO + O + A	BPPALND	O	—	—	N	5	5	TPN	—	—
		49	Ovary	Borderline serous tumor	Ia	TAH + BSO + O + A	BPPALND	O	—	—	N	6	5	TPN	—	—
		38	Ovary	Borderline serous tumor	Ic	TAH + BSO + O + A	BPPALND	O	—	—	N	20	60	MCT	—	—
		63	Endometrium	Endometrioid	Ia	TAH + BSO + O + A	BPPALND	O	—	—	N	39	29	MCT	—	—
		28	Ovary	Borderline serous tumor	Ic	RSO + O + A	BPPALND	NS	—	—	N	33	58	TPN	—	Y
		38	Ovary	Borderline serous tumor	Ia	RSO + myomectomy + O + A	BPPALND	NS	—	—	N	19	16	MCT	—	—
		53	Cervix	Adenosquamous carcinoma	Ib1	RH + BSO	BPPALND	O	Y	—	N	3	27	TPN	—	Y
		18	Ovary	Dysgerminoma	IIIc	RSO + O	BPPALND	NS	—	Y	N	8	41	TPN	—	—
		46	Ovary	Serous papillary cystadenocarcinoma	IIIc	TAH + BSO + O + A	BPPALND	O	—	Y	N	40	35	MCT	—	—
		28	Ovary	Sertoli–Leydig cell carcinoma	Ic	RSO + O + A	BPPALND	NS	—	—	N	13	42	TPN	—	Y
		32	Ovary	Granulosa cell carcinoma	Ia	RSO + O + A	BPPALND	NS	—	—	N	13	41	TPN	—	Y
		31	Ovary	Borderline mucinous tumor	Ia	TAH + BSO + A + omental biopsy	BPPALND	O	—	—	N	12	83	MCT + TPN	Somatostatin	Y
		61	Ovary	Serous papillary cystadenocarcinoma	IIIc	TAH + BSO + O + A	BPPALND	O	—	—	N	41	23	TPN	—	Y
		38	Ovary	Borderline serous tumor	Ic	TAH + BSO + O + A	BPPALND	O	—	—	N	27	21	TPN	—	—
		44	Ovary	Serous papillary cystadenocarcinoma	IIIc	TAH + BSO + O + A	BPPALND	O	Y	Y	Chemotherapy	14	27	MCT	—	—
		34	Ovary	Malignant struma ovarian	Ic	RSO + O + A	BPPALND	NS	—	—	N	7	66	TPN	—	—
		66	Endometrium	Endometrioid	Ib	TAH + BSO + omental biopsy	BPPALND	O	—	—	N	14	32	MCT	—	—
		22	Ovary	Dysgerminoma	IIIc	LSO + right salpingectomy + O + A	BPPALND	NS	Y	Y	N	9	11	MCT	—	—
		32	Ovary	Borderline serous tumor	Ic	RSO + O + A	BPPALND	NS	—	—	N	2	6	TPN	—	—

Frey et al.	2012	58	Endometrium	Endometrioid adenocarcinoma	IB	TAH + BSO + O + A	BPPALND	O	NS	NS	N	9	90	MCT	—	—
		52	Endometrium	Mucinous adenocarcinoma	IB	TAH + BSO + O + A	BPPALND	O	NS	NS	N	18	44	MCT	—	—
		69	Endometrium	Endometrioid adenocarcinoma	IA	TAH + BSO + O + A	BPPALND	O	NS	NS	N	12	32	MCT	—	—
		61	Endometrium	Serous adenocarcinoma	IC	TAH + BSO + O + A	BPPALND	O	NS	NS	N	0	9	MCT	—	—
		46	Endometrium	Endometrioid adenocarcinoma	IIB	TAH + BSO + O + A	BPPALND	O	NS	NS	N	22	12	MCT	—	—
		68	Endometrium	Endometrioid adenocarcinoma	IB	TAH + BSO + O + A	BPPALND	O	NS	NS	N	11	69	MCT	—	—
		65	Endometrium	Endometrioid adenocarcinoma	IB	TAH + BSO + O + A	BPPALND	O	NS	NS	N	8	2	MCT	—	—
		53	Endometrium	Endometrioid adenocarcinoma	IIIC	TAH + BSO + O + A	BPPALND	O	NS	NS	N	20	23	MCT	—	—
		79	Endometrium	Malignant mixed Müllerian tumor	IIC	TAH + BSO + O + A	BPPALND	O	NS	NS	N	13	36	MCT	—	—
		42	Endometrium	Endometrioid adenocarcinoma	IIIC	TAH + BSO + O + A, colon resection	BPPALND	O	NS	NS	N	6	24	MCT	—	—
		67	Primary peritoneal	Serous adenocarcinoma	IV	RH + O + A, colon resection	BPPALND	O	NS	NS	N	17	14	MCT	—	—
		51	Breast metastatic to ovary	Invasive ductal breast carcinoma	IV	TAH + BSO	P	O	NS	NS	N	18	54	MCT	—	—

Zhao et al.	2014	55	Endometrium	Adenocarcinoma	IIIc1	TH + BSO	BPPALND	L	NS	NS	N	9	9	TPN	Somatostatin	—
		55	Cervix	Mucinous adenocarcinoma	IB1	RH + BSO	P	L	NS	NS	N	4	6	MCT	—	—
		52	Endometrium	Adenocarcinoma	IB	TH + BSO	BPPALND	L	NS	NS	N	2	4	MCT	—	—
		42	Ovary	Mucinous adenocarcinoma	IA	TH + BSO + O + A	P	L	NS	NS	N	3	4	MCT	—	—
		43	Endometrium	Carcinosarcoma	IA	TH + BSO + O	BPPALND	L	NS	NS	N	4	9	TPN	Somatostatin	—
		58	Endometrium	Adenocarcinoma	IB	TH + BSO	BPPALND	L	NS	NS	N	4	3	MCT	—	—
		45	Cervix	Squamous cell carcinoma	IB1	RH	BPPALND	L	NS	NS	N	4	7	MCT	—	—
		55	Endometrium	Adenocarcinoma	IA	TH + BSO	BPPALND	L	NS	NS	N	4	7	MCT	—	—
		42	Cervix	Squamous cell carcinoma	IB1	RH	BPPALND	L	NS	NS	N	3	7	MCT	—	—

Göçmen et al.	2014	58	Endometrium	Carcinosarcoma	IB	H + BSO	BPPALND	Robot-assisted L	—	—	N	14	7	MCT + TPN	Octreotide	—
		50	Endometrium	Adenocarcinoma	IB	H + BSO	BPPALND	Robot-assisted L	—	—	N	3	11	MCT + TPN	Octreotide	—
		54	Endometrium	ns	NS	H + BSO	BPPALND	Robot-assisted L	NS	NS	N	2	4	MCT		—
		56	Endometrium	Endometrioid adenocarcinoma	IB	H + BSO	BPPALND	Robot-assisted L	—	—	N	2	5	MCT + TPN	Octreotide	—

Kim et al.	2014	43	Endometrium	Endometrioid adenocarcinoma	NS	H	BPPALND	L	—	—	N	4	82	MCT + TPN	Octreotide	—

Solmaz et al.	2015	56	Ovary	Serous	IC	TAH + BSO + O	BPPALND	O	—	—	N	3	7	TPN	—	—
		68	Endometrium	Endometrioid	IB	TAH + BSO + O	BPPALND	O	—	—	N	4	3	MCT	—	—
		56	Endometrium	Clear cell	IV	RH + BSO + O + A	BPPALND	O	—	—	N	5	3	TPN	—	—
		47	Endometrium	Endometrioid	IIIC	TAH + BSO + O	BPPALND	O	Y	—	N	3	6	TPN	—	—
		60	Ovary	Serous	IIIC	TAH + BSO + O	BPPALND	O	—	—	N	5	3	TPN	—	—
		56	Endometrium	Endometrioid	IIIC	TAH + BSO + O	BPPALND	O	Y	—	N	3	3	MCT	—	—
		41	Ovary	Serous	IIIC	TAH + BSO + O	BPPALND	O	Y	Y	N	4	6	TPN	—	—
		64	Synchronous primary endometrial and ovarian cancer	Serous and endometrioid	IIIC	TAH + BSO + O	BPPALND	O	Y	Y	N	2	5	TPN	—	—
		68	Endometrium	Serous and endometrioid	IIIC	TAH + BSO + O	BPPALND	O	Y	—	N	3	5	MCT	—	—
		42	Synchronous primary endometrial and ovarian cancer	Serous and endometrioid	IV	RH + BSO + O + A	BPPALND	O	Y	Y	N	3	7	TPN	—	—
		40	Ovary	Mucinous	IC	TAH + BSO + O + A	BPPALND	O	—	—	N	6	2	MCT	—	—
		55	Ovary	Endometrioid	IA	TAH + BSO + O	BPPALND	O	—	—	N	5	7	TPN	—	—
		50	Ovary	Serous	IIIC	TAH + BSO + O, peritonectomy, hyperthermic intraperitoneal chemotherap	BPPALND	O	Y	Y	N	4	4	TPN	—	—
		53	Endometrium	Endometrioid	IB	TAH + BSO	BPPALND	O	—	—	N	3	3	TPN	—	—
		48	Endometrium	Endometrioid	IA	TAH + BSO	BPPALND	O	—	—	N	5	10	TPN	Somatostatin	—
		41	Endometrium	Endometrioid	IB	TAH + BSO	BPPALND	O	—	—	N	7	3	TPN	—	—
		68	Ovary	Endometrioid	IC	TAH + BSO + O	BPPALND	O	—	—	N	3	10	TPN	—	—
		62	Endometrium	Endometrioid and clear-cell carcinoma	IB	TAH + BSO + O	BPPALND	O	—	—	N	3	12	TPN	Somatostatin	—
		47	Endometrium	Malignant mixed Müllerian tumor	IB	TAH + BSO + O	BPPALND	O	—	—	N	4	4	TPN	—	—
		33	Synchronous primary endometrial and ovarian cancer	Endometrioid + endometrioid	IC-IA	TAH + BSO + O	BPPALND	O	—	—	N	4	3	TPN	—	—
		76	Synchronous primary endometrial and ovarian cancer	Serous and endometrioid	IIIC	TAH + BSO + O	BPPALND	O	—	Y	N	3	4	TPN	—	—
		62	Ovary	Squamous cell carcinoma	IIIC	TAH + BSO + O	BPPALND	O	—	—	N	5	7	TPN	Somatostatin	—
		55	Cervix	Adenocarcinoma	IB1	RH + BSO	BPPALND	O	Y	Y	N	4	9	TPN	Somatostatin	—
		47	Ovary	Serous	IIIC	TAH + BSO + O	BPPALND	O	—	—	N	5	5	TPN	Somatostatin	—
		48	Ovary	Serous	IIIC	RH + BSO + O + A	BPPALND	O	—	Y	N	3	6	TPN	—	—
		56	Ovary	Serous	IIIC	—	BPPALND	O	—	—	N	5	3	TPN	—	—
		59	Endometrium	Endometrioid	IB	TAH + BSO + O	BPPALND	O	—	—	N	3	5	TPN	—	—
		56	Endometrium	Endometrioid	IIIC	TAH + BSO + O	BPPALND	O	Y	—	N	4	3	TPN	—	—
		61	Endometrium	Serous	IB	TAH + BSO + O + A	BPPALND	O	—	—	N	4	10	TPN	—	—
		53	Ovary	Serous	IIIC	TAH + BSO + O + A	BPPALND	O	—	Y	N	3	9	TPN	—	—
		54	Fallopian tube	Endometrioid	IC	TAH + BSO + O	BPPALND	O	—	—	N	4	5	TPN	—	—
		48	Ovary	Serous	IA	O + A	BPPALND	O	—	—	N	4	4	TPN	—	—
		50	Endometrium	Clear-cell carcinoma	II	TAH + BSO + O	BPPALND	O	Y	Y	N	4	6	TPN	—	—
		53	Endometrium	Endometrioid	IIIC	TAH + BSO + O	BPPALND	O	—	—	N	4	7	TPN	—	—
		56	Endometrium	Endometrioid	IB	TAH + BSO + O	BPPALND	O	—	—	N	5	3	TPN	—	—
		44	Synchronous primary endometrial and ovarian cancer	Endometrioid + endometrioid	IA-IA	TAH + BSO + O	BPPALND	O	—	—	N	3	10	TPN	—	—
		49	Endometrium	Endometrioid adenocarcinoma	IB	TAH + BSO	P	O	—	—	N	NS	42	MCT	Octreotide	—

Thiel et al.	2016	40	Cervix	Squamous	I (UICC)	RH	BPPALND	L	—	—	N	53	10	TPN	—	—
		60	Cervix	Squamous	I	TLH + BSO	BPPALND	L	—	—	N	16	2	Observation	—	—
		45	Endometrium	Endometrioid	I	TLH + BSO	BPPALND	L	—	—	N	2	5	TPN	—	—
		70	Endometrium	Endometrioid	II	TLH + BSO	BPPALND	L	—	—	N	2	17	TPN	—	—
		54	Endometrium	Endometrioid	I	TLH + BSO	BPPALND	L	—	—	N	5	13	TPN	—	—
		45	Ovary	Serous	I	TAH + BSO + O	BPPALND	O	—	—	N	3	10	TPN	—	—
		38	Cervix	Squamous	II	RH	BPPALND	O	—	—	N	2	10	TPN	—	—
		41	Cervix	Squamous	IV	Laparoscopic staging	BPPALND	L	—	Y	N	5	4	MCT	—	—
		58	Cervix	Adenocarcinoma	II	RH + BSO	BPPALND	L	—	—	N	1	9	TPN	—	—
		52	Ovary	Serous	III	BSO + O	BPPALND	O	Y	—	N	1	9	TPN	—	—
		69	Ovary	Serous	III	BSO + O	BPPALND	O	—	—	N	5	12	TPN	—	—
		59	Endometrium	Endometrioid	III	TAH + BSO	BPPALND	O	—	—	N	3	10	TPN	—	—
		69	Endometrium		II	TLH + BSO	BPPALND	L	—	—	N	2	10	TPN	—	—
		63	Endometrium	Endometrioid	I		BPPALND	L	—	—	N	6	9	MCT	—	—
		42	Cervix	Squamous	I	TLH + BSO	BPPALND	L	—	—	N	4	6	TPN	—	—
		43	Cervix	Squamous	IV	Laparoscopic staging	BPPALND	L	—	Y	N	2	5	TPN	—	—
		63	Cervix	Adenocarcinoma	III	Laparoscopic staging	BPPALND	L	—	—	N	2	10	TPN	—	—
		22	Cervix	Adenocarcinoma	III	Laparoscopic staging	BPPALND	L	Y	—	N	2	5	TPN	—	—
		33	Cervix	Squamous	III	Laparoscopic staging	BPPALND	L	Y	—	N	1	4	TPN	—	—
		43	Cervix	Adenocarcinoma	II	RH	BPPALND	L	—	—	N	4	5	TPN	—	—
		47	Cervix	Squamous	III	Laparoscopic staging	BPPALND	L	Y	—	N	1	10	TPN	—	—
		57	Ovary	Serous	III	TAH + BSO + O	BPPALND	O	—	Y	N	3	9	TPN	—	—
		72	Endometrium	Endometrioid	II	TLH + BSO	BPPALND	L	—	—	N	1	10	TPN	—	—
		47	Cervix	Squamous	II	Laparoscopic staging	BPPALND	L	—	—	N	2	10	TPN	—	—
		83	Ovary	Clear cell	III	TAH + BSO + O	BPPALND	O	Y	—	N	8	2	TPN	—	—
		38	Cervix	Adenocarcinoma	I	RH	BPPALND	L	—	—	N	2	9	TPN	—	—
		68	Ovary	Serous	III	TAH + BSO + O	BPPALND	O	—	Y	N	3	3	MCT	—	—
		47	Endometrium	Endometrioid	III	TLH + BSO	BPPALND	L	—	—	N	2	4	MCT	—	—

Liu et al.	2017	23	Ovary	Dysgerminoma	NS	NS	RP	L	NS	NS	N	7	35	TPN	Somatostatin	Y

H: hysterectomy; O: open surgery; L: laparoscopy; MRH: modified radical hysterectomy; RH: radical hysterectomy; TAH: total abdominal hysterectomy; TH: total hysterectomy; LAVH: laparoscopically assisted vaginal hysterectomy; TLH: total laparoscopic hysterectomy; RT: radical trachelectomy; BSO: bilateral salpingo-oophorectomy; RSO: right salpingo-oophorectomy; LSO: left salpingo-oophorectomy; BPPALND: bilateral pelvic, para-aortic lymph node dissection; PA: para-aortic lymph node dissection; P: pelvic lymph node dissection; RP: right side pelvic lymph node dissection; O: omentum resection; A: appendix resection; TPN: total parenteral nutrition; MCT: high-protein, low-fat, medium chain triglyceride (MCT)-based diet; NS: not specified.

**Table 2:** Summary of patients' clinicopathological characteristics, surgical approach, and management.

Parameter (*n* = 140)	Number/range	Percentage (%)
Age	52 (18–83)	
Primary site		
Cervix	32	22.86
Ovary	46	32.86
Endometrium	55	39.29
Synchronous ovary and endometrium	5	3.57
Fallopian tube	1	0.71
Primary peritoneal	1	0.71
Surgical approach		
Open surgery	87	62.14
Laparoscopy	38	27.14
NS	15	10.71
Lymphadenectomy		
BPPALND	122	87.14
P	14	10.00
PA	3	2.14
No	1	0.71
Lymph node status		
Only positive pelvic LN	11	7.86
Only positive para-aortic LN	11	7.86
Positive para-aortic and pelvic LN	9	6.43
Negative para-aortic and pelvic LN	73	52.14
NS	36	25.71
Time to onset (days), median	5	0–75
Time to resolution (days), median	9	2–90
Management		
MCT	39	27.86
TPN	73	52.14
MCT + TPN	13	9.29
Observation	1	0.71
NS	14	10.00
Surgical treatment		
Yes	10	7.14
No	130	92.86
Medical therapy		
Somatostatin	21	15.00
No	103	73.57
NS	16	11.43

BPPALND: bilateral pelvic, para-aortic lymph node dissection; PA: para-aortic lymph node dissection; P: pelvic lymph node dissection; TPN: total parenteral nutrition; MCT: high-protein, low-fat, medium chain triglyceride (MCT)-based diet; NS: not specified.

**Table 3:** The median onset and resolution time in each group.

Parameter	Case	Time to onset (median days)	Time to resolution (median days)
*Primary site*			
Cervix	32	4 (1–69)	9 (2–53)
Ovary	44	6 (1–75)	9.5 (2–90)
Endometrium	53	4 (0–39)	9 (2–90)

*Surgical approach*			
Open surgery	84	5 (0–41)	9 (2–90)
Laparoscopy	38	3.5 (1–53)	8 (2–82)

*Lymphadenectomy*			
BPPALND	121	4 (0–53)	9 (2–90)
P	13	7 (3–69)	17 (4–54)
PA	2	40 (5–75)	15.5 (3–28)

*Lymph node status*			
Only positive pelvic LN	11	3 (1–22)	5 (2–27)
Only positive para-aortic LN	11	3 (2–75)	6 (3–41)
Positive para-aortic and pelvic LN	9	4 (2–14)	9 (4–27)
Negative para-aortic and pelvic LN	72	5 (1–69)	10 (2–90)

*Conservative management*			
MCT	—	—	12 (2–90)
TPN	—	—	9 (2–66)
MCT + TPN	—	—	18 (5–90)

*Surgical treatment*			
Yes	—	—	41 (16–90)
No	—	—	9 (2–90)

*Medical therapy*			
Somatostatin	—	—	10 (5–90)
No	—	—	9 (2–90)

BPPALND: bilateral pelvic, para-aortic lymph node dissection; PA: para-aortic lymph node dissection; P: pelvic lymph node dissection; TPN: total parenteral nutrition; MCT: high-protein, low-fat, medium chain triglyceride (MCT)-based diet.

## Data Availability

The original datasets used and/or analyzed during the current study are available from the corresponding author on reasonable request.
